# Red blood cell volume is not decreased in ESA‐naive anemic chronic kidney disease patients

**DOI:** 10.14814/phy2.13900

**Published:** 2018-11-13

**Authors:** Carsten Lundby, Belen Ponte, Anne‐Kristine Lundby, Paul Robach, Sophie de Seigneux

**Affiliations:** ^1^ Department of Clinical Medicine Rigshospitalet ‐ Finsencentret København Denmark; ^2^ Service and Laboratory of Nephrology Department of Internal Medicine Specialties and PHYME Department University Hospital of Geneva Geneva Switzerland; ^3^ Ecole Nationale des Sports de Montagne, site de l'Ecole Nationale de Ski et d'alpinisme Chamonix France

**Keywords:** Anemia, blood volume, chronic kidney disease, diuretic, plasma volume

## Abstract

Anemia is defined according to decreased blood hemoglobin concentration ([Hb]), which is considered a marker of low total red blood cell volume (RBCV). Alterations of plasma volume (PV) may also modify [Hb] without concomitant changes in RBCV. Since anemia and fluid retention are frequent complications of chronic kidney disease (CKD), we hypothesized that anemia during CKD may in part be related to expanded PV without a simultaneous decrease in RBCV. We quantified hemoglobin mass, RBCV, PV, and total blood volume (BV) using an automated carbon monoxide device in 40 consecutive stage 3–5 CKD patients not on dialysis and in seven healthy male controls of the same age range. These were compared within and to predicted volumes according to Nadler's formula. Arterial stiffness and NT‐proBNP were measured. RBCV was similar to predicted values range in anemic CKD patients 2073 (1818–2704) versus, 2061 (1725–2473) mL, *P* > 0.05. In contrast, PV was largely increased in anemic CKD patients (3881 (3212–4352) vs. 2916 (2851–3201)), *P* = 0.01. Of 26 anemic patients, only six had a >20% decrease in RBCV as the cause for their anemia, whereas 14 had a >20% increase of PV as a cause for their anemia. NT‐pro BNP correlated with eGFR but neither with PV nor BV, whereas arterial stiffness was not correlated to blood volumes. Anemia in CKD as diagnosed by low [Hb] is not necessarily associated to low RBCV but may reflect increased PV. This finding has implications for the treatment of CKD patients and may refrain from normalizing [Hb] levels in all CKD patients.

## Introduction

Anemia is defined as a hemoglobin concentration ([Hb]) <120 g/L in women and <130 g/L in men. The assumption is that a low [Hb], an easy parameter to measure, is equivalent to a reduced total volume of red blood cells (RBCV), which is more difficult to measure. In chronic kidney disease (CKD), recombinant erythropoietin (rhEpo) can be administrated to increase RBCV and thereby correct [Hb] toward “normal” values. While it is obvious that [Hb] is expected to decrease when RBCV is reduced, alterations in plasma volume (PV) may also influence [Hb]. In line with this a recent study demonstrates that hemoglobin mass (Hb_mass_; from which RBCV can be derived) does not correlated well with [Hb] in heart failure (HF) and cirrhosis patients (Otto et al. 2017). Anemic HF patients may exhibit low, normal, or even elevated RBCV, suggesting that in some cases the main cause for anemia is an expanded PV (Miller 2016). Even champion endurance athletes who possess very large RBCV may appear anemic due to an even greater increase in PV (Lundby et al. 2012). Anemia related to hemodilution is known as “pseudo anemia” (Bartsch et al. 1998). While [Hb] is a poor indicator for O_2_ transport and uptake, RBCV and/or Hb_mass_ correlate well herewith (Kanstrup and Ekblom 1984; Lundby and Robach 2015). Since fluid retention and PV expansion are highly prevalent with advanced CKD (Puri et al. 2014; Soi and Yee 2017), we hypothesized that the cause for anemia in CKD could partly be explained by an expanded PV rather than a reduced RBCV. If anemia can indeed, at least to some extend be attributed to PV expansion in CKD patients, this could have direct implications for treatment considering the adverse side effects related to rhEPO therapy (Pfeffer et al. 2009; Levin and Beaulieu 2010) and a more critical view on [Hb] values may be proposed.

Evaluation of plasma volume and hypervolemia is not available in all clinical settings. ANP, BNP, and NT‐Pro BNP are considered as markers of hypervolemia and are extensively used clinically for their prognostic value. NT‐Pro BNP has been described to be associated to eGFR during CKD, as well as to left ventricular myocardial hypertrophy (Colbert et al. 2015). NT‐proBNP is also associated to hard outcomes such as death and CV events in CKD patients (Horii et al. 2013). Whether NT‐pro BNP is a good reflection of increased plasma volume in CKD is unknown.

Pulse wave velocity (PWV) is a classical technique measuring arterial stiffness (Cavalcante et al. 2011) and has been associated with cardiovascular events (Vlachopoulos et al. 2010). Arterial stiffness is strongly associated to red blood cell volume in healthy individuals, suggesting that arterial stiffness could modify erythropoiesis (Montero et al. 2016). Whether such a correlation exist in patients with kidney disease, who usually have increased arterial stiffness, and could participate to anemia is unknown (Briet et al. 2006).

In this study, we quantified Hb_mass_ in anemic and nonanemic CKD patients using an automated device and derived their RBCV, PV, and total blood volume. The aim was to compare measured to predicted values in anemic, nonanemic, and controls. We further wished to study the predictive value of NT‐pro BNP and arterial stiffness for blood volume measures.

## Methods

### Patients

We recruited 50 consecutive stage 3–5 CKD patients from the Nephrology clinic at the University Hospital of Geneva, not on dialysis. Patients were stable outpatients, without modifications of diuretics prescription in the previous month and not treated with rhEpo. Ten patients were excluded from analysis due to incomplete data sets. The causes for CKD were diverse with a majority of diabetic/hypertensive nephropathies, followed by glomerular nephropathies and tubulo‐interstitial disease. Seven healthy men without renal disease were included as controls. All participants signed an informed consent. The study was approved by the ethical committee of the canton of Geneva (CER 2017‐00421) and performed according to the Declaration of Helsinki principles.

### Hemoglobin mass and blood volumes

Hemoglobin mass (Hb_mass_) was quantified using an automated system (OpCO, Detalo Instruments, Denmark) as previously described (Dandanell et al. 2016; Montero et al. 2017). We have previously demonstrated that obtaining RBCV, PV, and BV using the 10 minute carbon monoxide (CO) rebreathing method is a valid approach (Keiser et al. 2017). Ideal blood volumes were calculated according to Nadler and by setting htc to theoretical values of 44.5 for males and 39% for females (Nadler et al. 1962).

Erythropoietin was measured using an ELISA Kit (R&D Systems, Minneapolis, MN). NT‐ProBNP was measured on serum using Roche Cobas 8000 method ECLIA.

### Pulse wave velocity

We used PWV to assess arterial stiffness. Measures were made using the Sphygmocor Xcel (ATCOR Medical Inc, Sidney. Australia). After 15 min of rest in the supine position, an applanation tonometer was applied at the carotid artery and a thigh cuff on the femoral to measure carotid‐femoral PWV in m/sec. A cuff was also placed on the arm to measure central blood pressure and augmentation index (AI) using a validated transfer function.

### Statistical analysis

We describe median and interquartiles range (IQR: 25th to 75th percentiles) for all variables. We compared anemic and nonanemic CKD men to controls using Krusskall–Wallis test. Post‐hoc analyses were performed with Wilcoxon ranksum test. For multiple comparisons, we applied Bonferroni correction with a *P*‐value <0.02. Paired wilcoxon test was used to compare predicted and measured values in the same patients. For correlations Pearson's test was performed.

## Results

### CKD patients and control volunteer characteristics

Characteristics of all the participants are described in Table 1, separately for men and women. Among CKD patients (35 men, 5 women), median age was 66 (IQR 56–70) years, eGFR 31 (26–37.5) mL/min/1.73 m^2^. According to [Hb] for males (119; 111–130 g/L) and females (122; 121–122 g/L), 26 of 40 CKD patients were considered anemic according to the classical definition (Hb < 130 g/L for men, <120 g/L for women). [Hb] in controls was 147.8 (141.1–151.1) g/L, and none was anemic**.**

**Table 1:** Characteristics of all included CKD patients and controls and comparison between anemic and nonanemic males

Variables	ALL CKD *n* = 40	CKD men *n* = 35	CKD women *n* = 5
Anthropometrical			
Age	66 (56–70)	67 (58–70)	44 (43–54)
Weight kg	85.4 (73.2–100.3)	88.0 (77.0–100.6)	71.5 (60.8–73.3)
Height cm	171. 5 (167.5–179.0)	172 (168–180)	152 (151–169)
Renal function			
eGFR mL/min/1.73 m^2^	31 (26.0–37.5)	31 (26–40)	30 (29–31)
UACR mg/mmol	69.9 (11.2–222.9)	97.4 (10.6–220.2)	28.2 (18.3–2225.6)
Blood parameters			
[Hb] (g/L)	121.5 (112.5–129.5)	119 (111–130)	122 (121–122)
Epo IU/mL	10.7 (8.4–16.7)	10.8 (8.4–16.0)	10.7 (10.4–18.0)
Hb mass g	699 (594.5–852.5)	770 (625–866)	510 (491–538)
Hb mass g/kg	8.0 (7.2–9.1)	8.2 (7.2–10.1)	7.0 (6.9–7.6)
Measured BV (mL)	5897.5 (4975.5–6812.5)	6159 (5389–6978)	4016 (3990–4448)
*Predicted BV* (*mL*)	*5241.5* (*4627.5–5797.0*)	*5304* (*4753–5820*)	*4067* (*3323–4251*)
Difference BV (%)	11.0 (0–21.0)	11.5 (3.0–21.0)	4 (−1 to 17)
Measured RBCV (mL)	2121.0 (1832.5–2775.0)	2343 (1905–2859)	1536 (1511–1701)
*Predicted RBCV* (*mL*)	*2161.5* (*1736.0*–*2523.0*)	*2203* (*1923*–*2604*)	*1627* (*1329*–*1700*)
Difference RBCV (%)	9 (−5 to 17)	9 (−3 to 18)	0 (−7 to 13)
Measured PV (mL)	3747.0 (3065.5–4304.5)	3839 (3212–4336)	2607 (2505–2747)
*Predicted PV* (*mL*)	*2882.5* (*2565.5–3198.0*)	*2917* (*2614*–*3201*)	*2440* (*1994*–*2550*)
Difference PV (%)	19.0 (9.5–33.0)	21.0 (11.0–33)	7 (3–19)

eGFR, estimated glomerular filtration rate (CKD‐EPI); Hb, hemoglobin; EPO, serum erythropoietin BV: blood volume; RBCV, red blood cell volume; PV, plasma volume. All values are given in median and interquartile range (IQR). Differences are the ratio between (measured values‐predicted values) on predicted values in %. Predicted values are in italic and calculated according to Nadler's formula.

### Comparison between observed and predicted volumes in anemic and nonanemic patients

**Figure 1:**
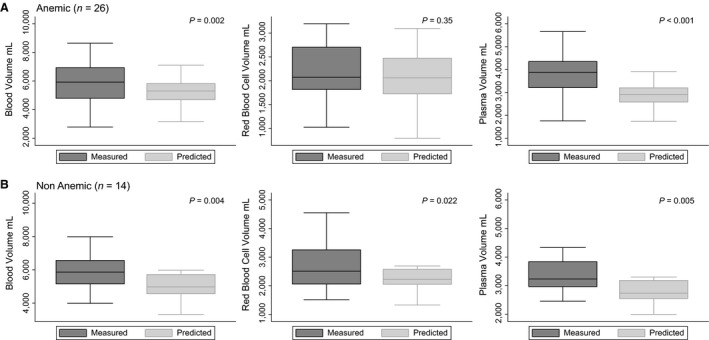
Boxplots of measured versus predicted volumes in anemic (A) and Nonanemic (B) CKD patients. Median and Boxplots comparing measured to predicted blood volumes in anemic (A) and nonanemic (B) patients. *P* values for the comparison between measured and predicted volumes are shown on each graphic. The horizontal bar inside each box is the median, the top and bottom of the box indicate the interquartile range, the T bars indicate the 95th percentiles.

We compared measured versus predicted blood values according to Nadler's formula, which are dependent on gender, weight and height, in either anemic or nonanemic CKD patients (Nadler et al. 1962). Boxplots comparing measured to predicted volumes values are displayed in Figure 1.

The anemic (*n* = 26) and nonanemic (*n* = 14) CKD patients did not differ by age, eGFR or albuminuria (*P* > 0.05). When comparing measured to predicted BV values, both anemic [5924 mL (4793–6930) vs. 5301 (4692–5820)] and nonanemic [5867 (5158–6562) mL vs. 4975 (4563–5718) mL] patients displayed elevated BV, confirming hypervolemia. Nonanemic patients also displayed higher measured than predicted RBCV [2511 (2063–3256) mL vs. 2225 (2053–2573) mL] and higher PV [3234 (2966–3839) mL vs. 2737 (2550–3182) mL]. Thus, in nonanemic CKD patients, PV and RBCV were expanded proportionally. In contrast, in the 26 anemic patients measured RBCV was not different from predicted values [2073 (1818–2704) mL vs. 2061 (1725–2473) mL] although measured PV was higher than predicted [(3881 (3212–4352) mL vs. 2916 (2581–3201) mL]. In control participants (Table 2), all measured values were similar to predicted ones for BV, RBCV, and PV (*P* > 0.05).

**Table 2:** Comparisons between anemic and nonanemic males and controls

Variables	Control men *n* = 7	Nonanemic CKD men *n* = 10	Anemic CKD men *n* = 25
Anthropometrical			
Age	62.0 (59–68)	67.5 (45–71)	66 (59–69)
Weight kg	77.0 (72.1–83.5)	87.2 (81.0–99.9)	88 (77–105)
Height cm	178 (172–184)	171.5 (167.0–176.0)	173 (170–180)
Renal function			
eGFR mL/min/1.73 m^2^	n/a	34 (24–49)	31 (26–28)
UACR mg/mmol	n/a	29.7 (12.4–163.4)	100.5 (6.0–277.5)
Blood parameters			
[Hb] (g/L)	147.8 (141.1–151.1)	143 (133–148)	115 (109–124)*#
Epo IU/mL	n/a	9.7 (8.5–12.2)	11.6 (8.4–19.6)
Hb mass g	800 (714–823)	836.5 (795.0–1070.0)	670 (598–837)#
Hb mass g/kg	10 (9.9–10.4)	10.9 (8.5–11.9)	7.5 (7.2–8.8)*#
Measured BV (mL)	5261 (5128–5784)	6226 (5814–7481)*	5973 (5319–6930)
* Predicted BV* (*mL*)	*5361* (*4665*–*5491*)	*5147* (*4920*–*5786*)	*5304* (*4753*–*5820*)
Difference BV (%)	2 (−2 to 2)	15.5 (9.0–23.0)*	10.5 (‐2 to 21)
Measured RBCV (mL)	2325 (2079–2435)	2633.5 (2484–3291)*	2090 (1847–2704)#
*Predicted RBCV* (*mL*)	*2413* (*2099*–*2471*)	*2316* (*2214*–*2604*)	*2075* (*1747*–*2473*)
Difference RBCV (%)	−1 (−4 to −1)	14.5 (4–21.0)*	8 (−12 to 14)
Measured PV (mL)	3090 (2936–3349)	3528.5 (3196–4189)*	3882 (3379–4352)*
*Predicted PV* (*mL*)	*2949* (*2566*–*3020*)	*2831.0* (*2706*–*3182*)	*2917* (*2614*–*3201*)
Difference PV (%)	6 (−1 to 17)	19 (9–26)*	22 (13–33)*

eGFR, estimated glomerular filtration rate (CKD‐EPI), Hb, hemoglobin, EPO, serum erythropoietin; BV, blood volume; RBCV, red blood cell volume; PV, plasma volume. All values are given in median and interquartile range (IQR). Differences are the ratio between (measured values‐predicted values) on predicted values in %. Predicted values are in italic and calculated according to Nadler's formula.

When comparing control males to anemic or nonanemic CKD patients, Kruskal–Wallis test was used, followed by Wilcoxon rank‐sum test: *P* < 0.016 compared to controls*, and compared to nonanemic CKD males^#^.

### Comparisons of volumes between men with CKD and controls

As controls only included males, we further compared male anemic and nonanemic CKD and controls in Table 2. Hb_mass_ was higher in nonanemic than anemic males, *P* < 0.01. Compared to control subjects, nonanemic CKD patients displayed both an increase in RBCV and BV (*P* < 0.01), while anemic patients revealed comparable BV and RBCV. PV difference was higher in anemic (*P* = 0.01) but not in nonanemic (*P* = 0.09) patients, compared to controls.

**Figure 2:**
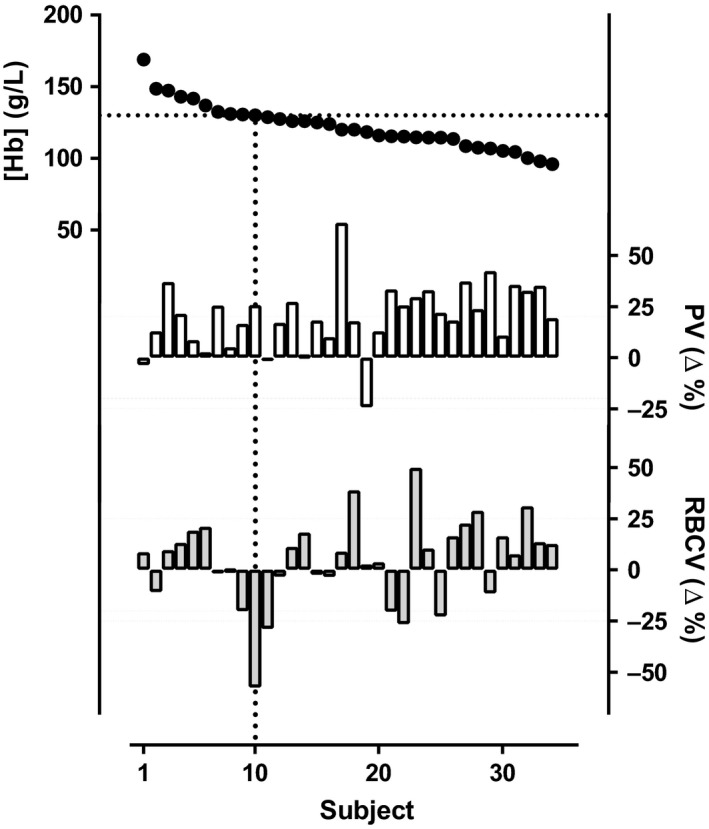
PV and RBCV as explanatory variables for anemia. Hemoglobin concentration (g/L; black circles), deviation (%) from idealized plasma volume (PV (%); white bars), and deviation (%) from idealized red blood cell volume (RBCV (%); gray bars) in nonanemic (left from vertical dotted line) and anemic (right from vertical dotted line) CKD patients. Since anemia is defined differently in male ([Hb] <130 g/L) and females ([Hb] <120 g/L) only male participants are shown in the figure for didactic purposes. The gray scaled area in PV and RBCV represents ±20% of idealized volume. From the figure it can be seen that anemia in CKDs may be manifested without affecting RBCV.

The extent of PV expansion and RBCV decrease was very variable at the individual level as shown in Figure 2. More specifically, in 6 of the 26 anemic patients anemia was related entirely to a lower than predicted (≥20% below predicted) RBCV while PV was within the normal range. In a further six anemic patients, anemia was related to the combination of less than a 20% reduction in RBCV with a less than 20% expanded PV. The remaining 14 anemic patients displayed markedly expanded (≥20% above predicted normal) PV while RBCV was within the expected range. We did not observe any correlation between the extent of PV increase and either eGFR or albuminuria.

Among our patients, 15 were diabetics and 25 were not. When comparing the difference between the predicted and measured PV, no significant difference was noted between diabetic (855 (657–1419) mL) and nondiabetic patients (634 (196–1306), *P* = 0.12., neither in absolute values nor as percent (*P* = 0.18).

### NT‐proBNP was not associated to blood and plasma volume

**Figure 3:**
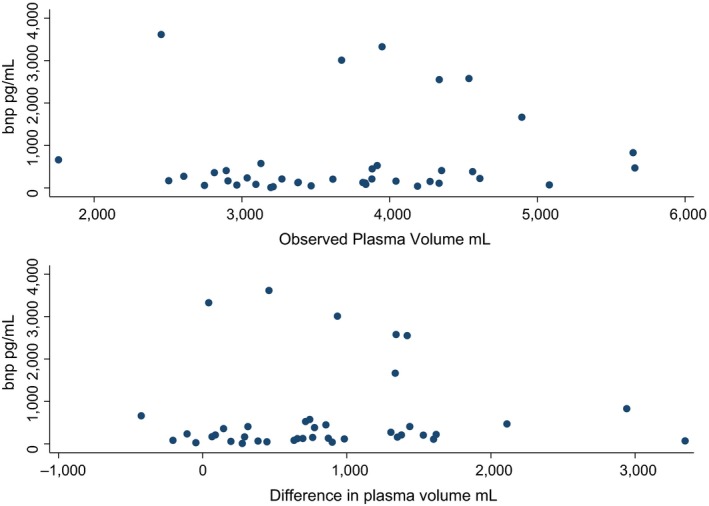
NT‐pro BNP and PV. Scatterplot analysis of NT‐proBNP and measured PV and delta PV.

NT‐proBNP was measured in all CKD patients. NT‐proBNP levels were not different between anemic and nonanemic CKD patients: 317 (152–327) versus 122 (67–236) pg/mL, *P* = 0.10. NT‐Pro‐BNP was significantly correlated to eGFR (*R* = −0.33, *P* < 0.05), whereas it was not to BV, PV or RBCV (*P* > 0.05 for all). NT‐Pro BNP did also not correlate with the differences between predicted and observed values for all measured volumes (PV, BV, RBCV), *P* > 0.05 for all (Fig. 3). Finally, NT‐proBNP did not correlate to arterial stiffness, neither using carotid‐femoral PWV nor AI (*P* > 0.05).

### Arterial stiffness was not associated to modifications of blood volumes

Arterial stiffness as assessed by AI or carotido‐femoral PWV was similar between anemic and nonanemic CKD patients: 24 (18–34) versus 29 (22–35) % for augmentation index and 10.1 (9.2–12.5) versus 10 (9.6–11.4) m/sec for carotid‐femoral PWV), *P* > 0.05. Neither AI, nor carotid‐femoral PWV were correlated to any of the measured blood volumes (PV, BV, RBCV) nor to the differential between each of the measured and the expected values, *P* > 0.05.

## Discussion

In this study, we quantified Hb_mass_ in anemic and nonanemic CKD patients as well as in healthy and age matched controls. From Hb_mass_ we deducted RBCV, PV, and BV and demonstrated that in the majority of anemic patients (20 out of 26, 77%), anemia was not related to a reduced RBCV, as expected, but rather to an excessively expanded PV. This suggests that [Hb] based diagnosis and treatment of anemia should be carefully considered.

The main outcome of the present study are blood volumes. Therefore, the technique used to quantify those volumes needs to be considered. CO rebreathing is a validated technique to determine RBCV, PV, and BV, and has been used extensively in physiological studies for more than a century (Thomsen et al. 1991; Siebenmann et al. 2017). More recently CO rebreathing has also been used in patient groups including heart failure (Karlsen et al. 2016) and cirrhotic patients (Otto et al. 2017). In the present study, we made use of the fully automated OpCO device which has a reported typical error of less than 1.5% (Dandanell et al. 2016; Fagoni et al. 2018). In a previous study, we measured a 3.1% reduction in Hb_mass_ as induced by 183 mL whole blood donation leading to a 3% decrease in Hb_mass_ (Keiser et al. 2017). It thus seems unlikely that the present results are caused by a measuring artifact. It should also be noted that RBCV, PV, and BV for all control subjects were within the predicted ranges and that CKD patients are known to retain fluids (Soi and Yee 2017).

In the present study we demonstrate that RBCV, a parameter better associated to O_2_ transport than [Hb] (Martino et al. 2002), is not decreased in anemic CKD patients, while PV is largely increased. This suggests that the cause for anemia in the majority of the investigated patient population is related mainly to hemodilution and not to a limited erythropoiesis, as also suggested by the similar erythropoietin values. Furthermore, our results indicate large interindividual variations in the distribution of RBCV and PV in CKD, suggesting that [Hb] should be considered carefully when diagnosing anemia in this population. Our findings that [Hb] based anemia, over‐diagnosed anemia in 20 of the 26 anemic patients is in line with previous observations suggesting that peripheral hematocrit determination may over‐diagnose anemia in 47% of hypervolemic patients (Kassebaum et al. 2014). The majority of our patients were non diabetic. When comparing diabetic and nondiabetic patients, the difference in PV was not different. We can, however, not exclude that this was also due to a lack of power.

Dilution is thus an important factor in the anemia of CKD patients. It may hence be speculated that by normalizing [Hb] with an erythropoietic stimulating agent (ESA) RBCV may increase to levels higher than normal. This may explain why normalizing [Hb] levels using rhEPO is not suitable for all patients and may contribute to the increased risks of stroke and arterial and venous thrombosis (Pfeffer et al. 2009). In line herewith, the beneficial effect of empaglifozin on cardiovascular mortality are apparently mostly linked to its diuretic effects and associated changes in [Hb] and htc, likely related to a decrease in plasma volume (Inzucchi et al. 2018). Our study may hence suggest that in pseudo anemic CKDs lowering PV by diuretic could be considered as a better therapeutic option than increasing RBCV.

While the direct determination of blood volumes is straight forward not all institutions may have access hereto. Hence, we aimed to establish weather a routine marker for PV such as NT‐proBNP can be used as a surrogate measure to distinguish between anemia and pseudo‐anemia in CKDs. Although NT‐proBNP displayed a tendency to be higher in anemic patients, and hence in line with expanded PV, it was not well‐correlated with neither PV nor BV. NT‐proBNP was correlated with renal function, as expected and previously described (Colbert et al. 2015). This suggests that NT‐proBNP is not a pure marker of volemia, but rather likely reflects eGFR, modification of heart structure in addition to volemia. In a similar manner, we attempted to determine whether arterial stiffness could be used as a tool to hint toward changes in RBCV. However, in contrast with our previous studies in healthy young volunteers where arterial stiffness is related to anemia and may decrease Hb production by regulating Epo production, arterial stiffness did not correlate with blood volumes in the current study (Montero et al. 2016).

Altogether, observed anemia in CKD may not strictly be related to diminished erythropoiesis and an according low RBCV but rather to elevated plasma volumes, which is secondary to abnormal sodium and fluid retention. In such patients, [Hb] levels may thus be misleading and should be interpreted with cautions when diagnosing anemia.

We encourage the assessments of RBCV and PV in future large scale clinical studies and call for care and blood volume evaluation in trials aiming at [Hb] normalization by ESAs.

### Physiological relevance

The role of volume retention on the anemia of chronic kidney disease has largely been underestimated. By applying established physiological measures of blood volumes we demonstrate that an augmented plasma volume contributes to the anemia of CKD and we suggest that treatment strategy should take this into account.

## Conflict of Interest

The authors report no conflict of interest.
